# A Brief Measure of Interpersonal Interaction for 2-Player Serious Games: Questionnaire Validation

**DOI:** 10.2196/12788

**Published:** 2019-07-23

**Authors:** Maja Gorsic, Joshua D Clapp, Ali Darzi, Domen Novak

**Affiliations:** 1 Department of Electrical and Computer Engineering University of Wyoming Laramie, WY United States; 2 Department of Psychology University of Wyoming Laramie, WY United States

**Keywords:** attitude to computers, competitive behavior, exercise, motivation, questionnaire design, virtual reality

## Abstract

**Background:**

Competitive and cooperative serious games have become increasingly popular in areas such as rehabilitation and education and have several potential advantages over single-player games. However, they are not suitable for everyone, and the user experience in competitive and cooperative serious games depends on many factors. One important factor is the verbal interaction between players, but the effect of this factor has not been extensively studied because of the lack of a validated measurement tool.

**Objective:**

This paper aimed to validate a brief questionnaire that measures the verbal interaction between 2 players of a serious game. The questionnaire consists of 8 questions pertaining to the amount of conversation, its valence (positive or negative emotion), and its game relatedness.

**Methods:**

The questionnaire was validated with 30 pairs of participants who played a competitive serious game for 10 min while being recorded with cameras. The questionnaire was filled out by both participants, an in-person observer, and 2 members of our research group who watched the videos. Results from these raters were used to develop questionnaire instructions, and the finalized questionnaire was given to 2 additional raters who were trained on 5 videos and then rated the other 25 videos independently.

**Results:**

The questionnaire’s interrater reliability is excellent for the amount of conversation and its game relatedness (intraclass correlation coefficients [ICCs] above 0.9). Interrater reliability is fair to good for conversation valence (ICCs between 0.4 and 0.7). We believe that the lower interrater reliability for valence is primarily because of a limited spread of valence values in our sample. Furthermore, questionnaire ratings were significantly correlated with players’ personality characteristics (eg, amount of conversation was correlated with extraversion) and pressure/tension experienced in the competitive game.

**Conclusions:**

The validated questionnaire has the potential to be a useful tool for studying user experience in competitive and cooperative serious games. Furthermore, it could be adapted for other applications such as entertainment games. However, it has only been validated with unimpaired university students in a 2-player competitive serious game and should next be validated with different target populations (eg, stroke survivors) and different game designs (eg, cooperative games).

## Introduction

### 2-Player Serious Games

Although serious games have traditionally involved only a single player, 2-player and multiplayer serious games have become increasingly popular in the last decade. For example, competitive and collaborative serious games can be used for motor rehabilitation [1-3]; for weight loss and general fitness [4-7]; for language therapy [8]; for military training [9]; to teach school subjects such as mathematics [10], language [11], and programming [12]; to teach more informal skills such as recycling [13], energy awareness [14], and sexual risk reduction [15]; and for many other applications. Such games have several potential advantages: compared with single-player games, competitive and cooperative games have been found to result in higher motivation and energy expenditure in rehabilitation and weight loss [3,4,16] as well as higher motivation and faster learning in educational applications [12].

However, not all users benefit equally from competitive and collaborative games. For example, studies in rehabilitation [2,17], weight loss [4], and education [13] have found that people who like competition often do not like cooperation (and vice versa). Furthermore, a person’s experience with a competitive or cooperative game depends on factors such as age, gender, personality, and the person’s relationship with the other player(s) [2,17-23]. The exact effects of these factors are often unclear and interaction effects are likely—for example, our previous studies on rehabilitation games have found that effects of personality are stronger in pairs of strangers than in pairs of friends [2,17]. To enable more effective deployment and personalization of serious games, these effects critically need to be studied in more detail.

### Interpersonal Interaction in 2-Player Serious Games

One factor that strongly affects users’ experience with competitive and cooperative serious games is the amount of interaction (both verbal and nonverbal) between players. For example, our previous study found a very strong correlation between self-reported enjoyment and the amount of conversation between 2 players [17], and other studies have found that self-reported enjoyment decreases as interaction elements between players are removed [20]. However, studying the effect of interpersonal interaction on user experience in serious games is difficult because a commonly accepted objective or subjective measure does not exist. In our previous study, we measured the amount of conversation between players using a simple 0 to 3 scale reported by an observer [16], but that measurement was not validated and had several methodological issues (eg, lack of consistent rating guidelines, no ability to rate positive vs negative conversation, and no ability to rate whether 1 player is talking more than the other). Other studies of 2-player serious games have acknowledged that verbal interaction between players is an important factor but did not analyze it because of a lack of a validated measure [1,3,24,25].

The goal of this study was to validate a brief questionnaire that measures the verbal interaction between 2 players of a serious game. The questionnaire consists of 8 questions pertaining to the amount of conversation, its valence (positive or negative emotion), and its game relatedness. The justification for these 3 quantities is as follows:

- Amount of conversation: previous research with unvalidated, ad-hoc measures has shown that the amount of conversation is correlated with subjective experience in competitive serious games [16] and that the amount of interaction differs between participant groups (eg, young and old [23]). Furthermore, although this has not been studied, we believe that the conversation balance (relative amount of talking done by each person in a pair) could provide insights into, for example, cooperation dynamics. Thus, measuring the amount of conversation is expected to provide insight into diverse aspects of competitive and cooperative serious games.
- Valence: providing a positive experience is a critical aspect of serious games for rehabilitation [1-3], physical fitness [7], and education [12], and we reasonably believe that a positive overall experience will result in positive conversational valence. Thus, a measure of conversational valence can provide insights into the players’ experience with the serious game.
- Game relatedness: although less well justified than the other 2 quantities, we believe that measuring game relatedness would provide important insights into whether the participants are focused on the serious game. Studies have suggested that participants need to be actively engaged with a serious game (rather than just going through the motions) to maximally benefit from it [26], and a high amount of game-unrelated conversation may indicate that the players are simply chatting rather than actively participating in the game.

The questionnaire is primarily meant to be filled out by an observer who watches the game session either in person or on video, although it could also be filled out by the players themselves. To validate the questionnaire, 30 pairs of participants played a competitive exercise game for 10 min while recorded on video. Results of the questionnaire are compared between the participants, an in-person observer, and several raters who watched the videos after the data collection had been completed.

## Methods

In this section, we first present the questionnaire and its rating instructions, then describe the methods used to validate the questionnaire.

### Questionnaire Items

Our brief interpersonal interaction questionnaire was developed to evaluate the verbal interaction between 2 game players who are ideally colocated (in the same room). It consists of eight 5-point items that can be answered in less than 5 min by either a player (after gameplay has concluded) or an observer (during or after gameplay). For an observer, the items are as follows:

1. How much did player A talk to player B? (1: little to no talking; 5: nearly constant talking)
2. How much did player B talk to player A? (same scale as above)
3. How balanced was the conversation? In other words, did both players talk about the same amount or did 1 player talk more than the other? (1: both players talked about the same amount; 3: 1 player talked moderately more; and 5: 1 player dominated the conversation)
4. How positive or negative were the things that player A said during the game? (1: very negative; 5: very positive)
5. How positive or negative were the things that player B said during the game? (same scale as above)
6. Were the things that player A said about the game or other unrelated topics? (1: mostly unrelated to the game; 5: mostly related to the game)
7. Were the things that player B said about the game or other unrelated topics? (same scale as above)
8. How would you rate the overall conversation between the players? (1: very negative; 5: very positive)

For a player, these questions were modified to refer to “you” and “the other player” instead of players A and B. Full copies of both versions of the questionnaire are available in Multimedia Appendix 1. For observers, players A and B should be defined within the context of the experiment to avoid confusion; in our study, for example, we defined player A as the person sitting on the left and player B as the person sitting on the right.

### Rating Instructions

Administering the questionnaire is relatively easy and should not take more than a few minutes. However, as some situations can be confusing for raters, the following instructions should be read carefully before using the questionnaire to ensure consistent answers.

*General*: Raters should answer all the questions even if the amount of conversation is limited; answers of “not applicable” should only be permitted if a player says nothing during the entire gameplay session. Raters are allowed to take notes and make preliminary scores during the gameplay interval and may rewind and replay the videos as desired. However, raters have to watch the entire interval before giving their final ratings.

*Items 1 and 2* (amount of conversation): If both players talk almost constantly, both should receive a score of 5; conversely, if 1 player talks for approximately half the gameplay session and the other never talks, the silent player should receive a score of 1, whereas the talking player should receive a 3 or 4.

*Item 3* (balance): Very high values (4 or 5) should only be used in cases where 1 player is talking frequently and the other is not. For example, if 1 player talks for 5 min and the other player never talks, that would be a 5. However, if 1 player never talks and the other player only says 1 sentence, that should be rated a 2.

*Items 4 and 5* (valence): Raters should rate all things spoken, not only those directed at the other person. For example, if a player appears to be talking to themselves, those comments should be considered for these items. Furthermore, raters should rate not only the words but also the tone and facial expression. For example, neutral words (eg, “the game is getting harder”) accompanied by a smile should result in a 4. In cases of sarcasm (eg, “you did such a good job” said insincerely), raters should consider the other player’s reaction—if the other player appears amused by the sarcasm, it should be treated as positive; conversely, if the other player appears annoyed by the sarcasm, it should be treated as negative. Finally, raters should use the extreme values (1 and 5) sparingly—1 should be used when the players are actively antagonizing each other, whereas 5 should be used when the players are actively praising each other or the game.

*Items 6 and 7* (game relatedness): The answers 1 and 5 indicate “mostly unrelated to the game” and “mostly related to the game”, respectively. The players do not need to talk 100% about the game to get a 5, and the answers 1 and 5 can be used relatively frequently here. For example, a 5 would correspond to about 90% of the conversation being game related, whereas a 1 would correspond to about 10% of the conversation being game related. Furthermore, metagame discussion (eg, criticizing the game's features or wondering how it is programmed) counts as game related. However, discussion about other games (other than the one being played) does not count as game related.

*Item 8* (overall mood): This item is not meant to be an average of items 4 and 5 and does not only include conversation but also includes facial expressions and body language. The answers 1 and 5 can be used more frequently here than on questionnaire items 4 and 5; for example, if the players are smiling and appear to be having a good time, but their conversation is mostly about neutral topics (eg, “oh, the game is getting harder”), raters could answer 4 to items 4 and 5, but 5 to item 8.

### Evaluation

#### Our Serious Game

**Figure 1:**
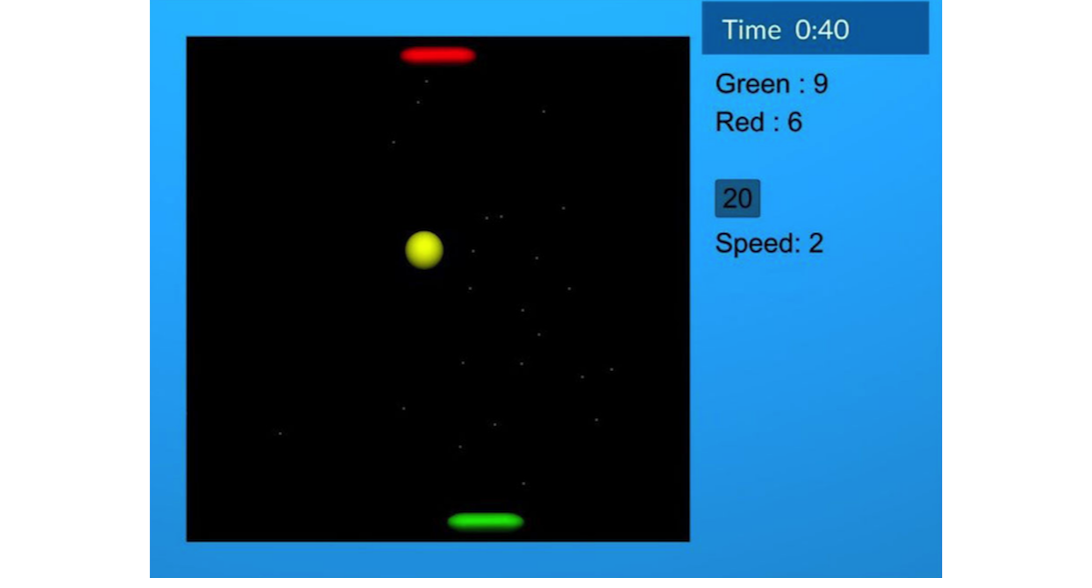
Screenshot of the competitive Pong game. Each player controls one of the 2 paddles. The current game duration, score, ball speed, and time until the next automated difficulty adaptation are shown on the right side of the playing field. Image reused from Gorsic et al with permission [16].

The validation study was performed with a single 2-player competitive serious game: the game of Pong previously used in our arm rehabilitation studies [16,17]. Each player controls a paddle near the top or bottom of the screen and moves it left or right using their controller. A ball bounces around the game field, and each player’s goal is to intercept the ball so that it does not pass their paddle. If the ball passes a player’s paddle and reaches the top or bottom of the screen, the opponent scores a point. Once the point is scored, the ball moves to the middle of the screen and begins moving in a random direction again after a 1-second pause. Every 60 seconds, the difficulty of the game changes according to a simple adaptation algorithm that changes the ball speed and the size of the 2 paddles depending on the players’ relative score as described in our previous paper [16]. A screenshot of the game is shown in Figure 1.

**Figure 2:**
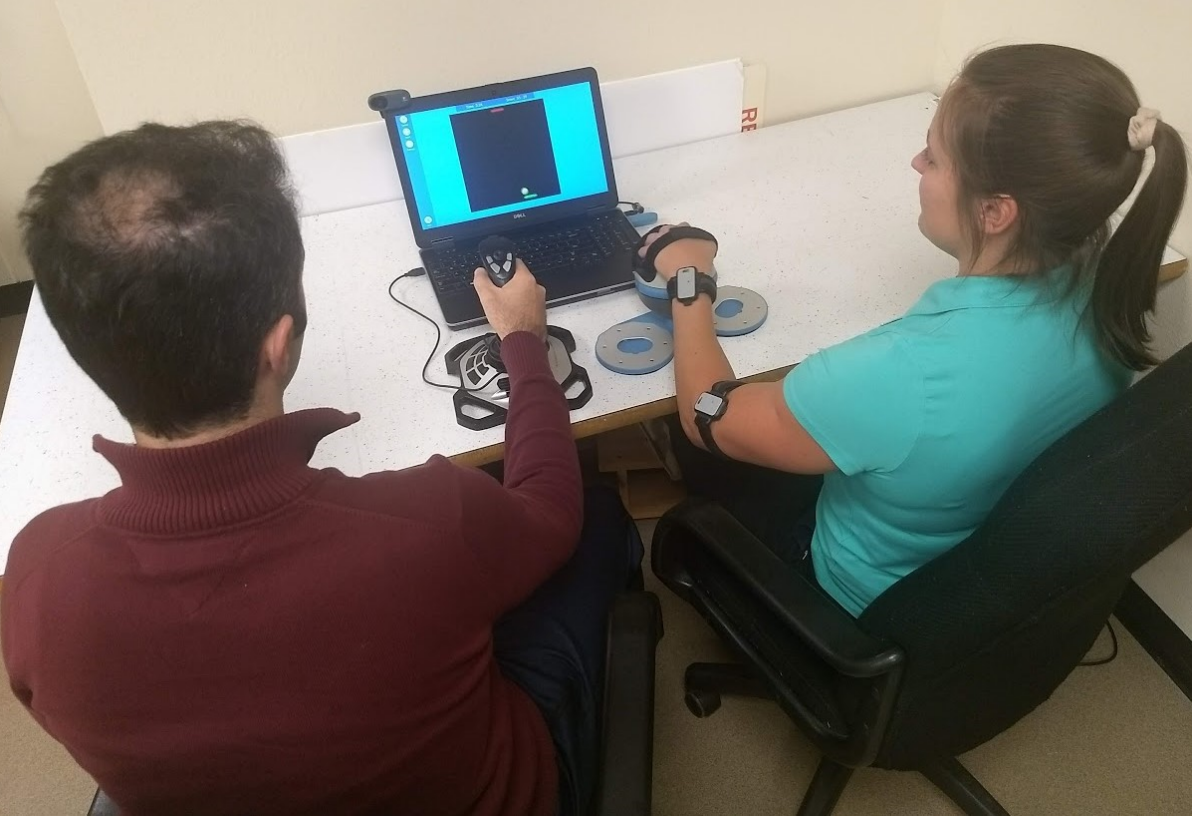
Study setup. Two participants play Pong using a joystick and Bimeo arm rehabilitation device while observed by an experimenter (not shown) and 2 webcams. Participants could use whichever arm they wished to play the game.

Both players play the game on the same computer and are seated side by side in front of the same screen. To control the game, we reused the same hardware from our previous rehabilitation study [16]. One player (*participant A*) uses a joystick and tilts it left and right to move their paddle left and right. The other player (*participant B*) uses a Bimeo arm rehabilitation device (Kinestica d.o.o), which consists of 2 acceleration sensors attached to armbands and a spherical handheld module; this module must be tilted left and right to move the player’s paddle left and right. A photo of 2 participants playing the game is shown in Figure 2.

#### Participants

Data collection was carried out in the first half of 2018. Overall, 30 pairs (60 participants) were recruited among students and staff of the University of Wyoming. Participants were not allowed to take part in the study if they had played our specific version of Pong before, although they were not asked if they had played Pong in general. Participants could volunteer for the session alone or in self-selected pairs (eg, 2 friends); if a participant volunteered alone, they were paired with another random single participant. No additional restrictions were placed on allowed pairings. Of the 30 pairs, 22 were same-gender pairs, whereas 8 were mixed-gender pairs; 14 were self-selected pairs, whereas 16 were paired together randomly. There were 42 male and 18 female participants (none self-identified as nonbinary), aged mean 22.3 (SD 5.5) years.

#### Study Protocol

**Figure 3:**
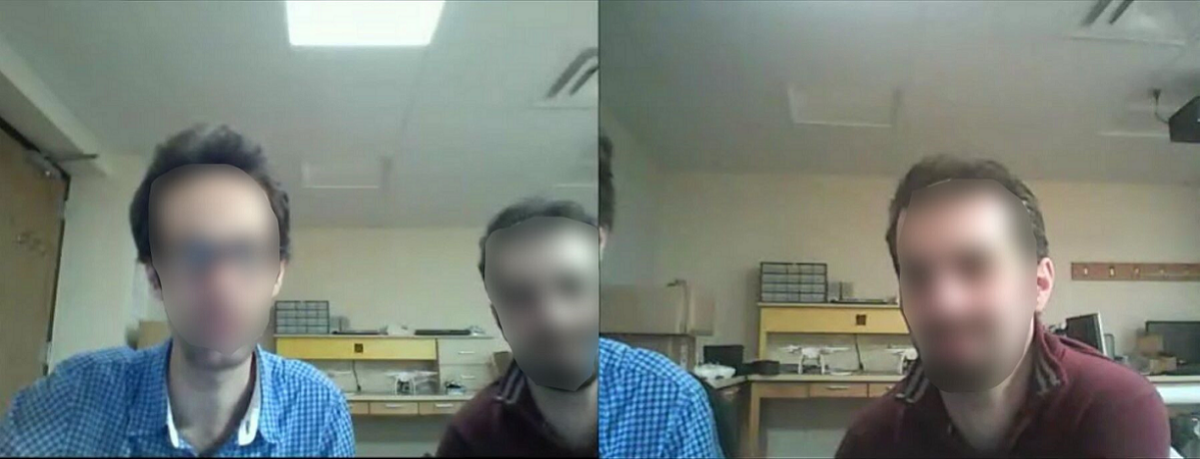
Screenshot of the video from the webcams. Videos from the 2 webcams are automatically synchronized and shown to raters side-by-side as one video. Faces are blurred for anonymity.

Each pair of participants took part in a single session. On arrival to the laboratory, participants were told that the purpose of the study was to examine player behavior in a competitive game and that they would be videotaped, although they were not specifically told that the goal was to validate the interpersonal interaction questionnaire. The experiment procedure was explained, the game and questionnaires were demonstrated, and informed consent forms were signed. Participants filled out a brief demographic questionnaire and were then seated in front of the computer and played the game for 10 min without breaks. A total of 2 webcams (1 atop the computer screen and 1 integrated into the screen) were used to take video and audio recordings of the participants. An example image from the 2 webcams is shown in Figure 3. After the 10-min period concluded, both participants filled out the interpersonal interaction questionnaire as well as an 8-item version of the Intrinsic Motivation Inventory (IMI), which measures 4 motivation scales during gameplay: interest/enjoyment, effort/importance, perceived competence, and pressure/tension, each using two 7-point Likert items. The specific version of the IMI was reused from our previous work [16]. Furthermore, they filled out the Ten-Item Personality Inventory, which measures the Big Five personality factors (extraversion, agreeableness, openness, conscientiousness, and neuroticism), each using two 7-point Likert items [27].

At the same time as the participants, the experimenter also filled out the interpersonal interaction questionnaire. The videos were then provided to 2 other raters (members of our research group), who filled out the interpersonal interaction questionnaire by watching the videos. Both raters scored each video, and the rating procedure was done in 2 stages to develop the rating instructions. The experimenter and the 2 video raters first collected and scored the first 15 videos. They then met to compare their answers, rewatched videos where the 3 ratings did not match (at least a 2-point discrepancy between any 2 raters on any item) and developed the rating instructions (*Rating Instructions* section) to address the discrepancies. With the rating instructions at hand, the experimenter and 2 raters adjusted their ratings for the first 15 videos as desired. After that, the remaining 15 pairs were rated independently by the experimenter and 2 video raters using the rating instructions.

Once all 30 pairs had been rated by the original 3 raters, the instructions were finalized, and the videos and the questionnaire were given to 2 external raters (paid US $10 per hour for this study, but otherwise unaffiliated with our research group). A total of 5 videos were selected as *training* videos: the external raters rated them one by one and received feedback about their score and clarifications about the rating procedure after each rated video. Of these 5 videos, the first 2 were considered *easy* by the original raters and had resulted in identical ratings; the other 3 videos were considered more difficult to rate and included either unbalanced conversation, an overall low amount of conversation (making it difficult to rate valence and game relatedness), or sarcasm (making it difficult to rate valence). For items 1-2 and 6-8, the external raters’ first ratings (before receiving feedback) were already very similar to those of the original raters for all 5 training videos. For item 3 (balance), 1 external rater had to be reminded that an answer of 1 corresponds to perfectly balanced conversation, as they had assumed that an answer of 3 corresponds to balanced conversation. For items 4 and 5 (valence), the external raters’ first ratings were very similar to the original raters’ first ratings for the first 3 training videos, but feedback was needed for the *low amount of conversation* and *sarcasm* training videos. After completing the 5 training videos, the external raters rated the other 25 videos independently.

#### Data Analysis

As the primary analysis, intraclass correlation coefficients (ICCs) were calculated between pairs of raters for each interpersonal interaction questionnaire item separately. ICCs are a standard method to assess the consistency of measurements made by different observers who are measuring the same quantity; in our case, they were calculated to determine whether our questionnaire can produce consistent ratings of the same conversation when used by different people, a necessary prerequisite for the use of the questionnaire in research. ICCs provide a more stringent test of consistency than standard Pearson correlations because their estimates account for both the covariation and absolute agreement between raters. They were calculated using a 2-way mixed, single-measures model with absolute agreement [28]. Interpretive benchmarks for ICCs (excellent: 0.75-1.00; good: 0.60-0.74; fair: 0.40-0.59; and poor: below 0.40) are provided by Cicchetti [29].

As the secondary analysis, we also calculated Spearman correlation coefficients between the interpersonal interaction questionnaire, the 4 IMI scales, and the Big Five personality factors. This provides an estimate of how the results of our questionnaire correlate with personality and game experience; as an important future application of the questionnaire is to study correlations between conversation and game experience in 2-player serious games, the secondary analysis provides preliminary data in this regard. For the interpersonal interaction questionnaire, the correlations were calculated separately using the averaged answers of the 3 original raters, the averaged answers of the 2 external raters, and the participants’ own answers.

For participants and the 3 original raters, ICCs and correlation coefficients were calculated over all 30 pairs; for external raters, only 25 pairs were included because the 5 training pairs were excluded. We chose to use all 30 pairs for the 3 original raters, as they updated their ratings after the rating instructions had been finalized, although we acknowledge that this may have introduced some bias.

## Results

Participants’ mean self-reported answers to the Ten-Item Personality Inventory (on scales of 2-14) were 10.6 (SD 1.5) for openness, 10.3 (SD 2.2) for conscientiousness, 8.2 (SD 2.7) for extraversion, 6.3 (SD 2.2) for agreeableness, and 5.8 (SD 2.2) for neuroticism. Their mean self-reported answers to the IMI (on scales of 2-14) were 10.3 (SD 2.5) for interest/enjoyment, 10.6 (SD 2.8) for effort/importance, 9.8 (SD 2.6) for perceived competence, and 7.3 (SD 3.1) for pressure/tension.

ICCs for different pairs of raters are shown in Table 1. Furthermore, Spearman correlation coefficients between the interpersonal interaction questionnaire, the IMI, and the Ten-Item Personality Inventory are listed in Tables 2 and 3.

**Table 1:** Intraclass correlation coefficients for the 8 questionnaire items and different pairs of raters.

Questionnaire item	Participants A-B	In-person^a^: video 1	In-person: video 2	Videos 1 and 2^b^	Externals 1 and 2^c^
1. How much A talks to B	0.82	0.92	0.91	0.92	0.94
2. How much B talks to A	0.75	0.93	0.93	0.91	0.94
3. Balance	0.27	0.63	0.47	0.45	0.63
4. Valence A to B	0.58	0.55	0.15	0.37	0.49
5. Valence B to A	0.65	0.59	0.42	0.48	0.65
6. Game relatedness A to B	0.51	0.93	0.93	0.97	0.90
7. Game relatedness B to A	0.75	0.98	0.93	0.94	0.97
8. Overall valence	0.52	0.60	0.69	0.65	0.68

^a^In-person: in-person observer (experimenter).

^b^Videos 1 and 2: video raters used to develop the questionnaire instructions.

^c^Externals 1 and 2: external video raters used for the validation of completed questionnaire.

**Table 2:** Spearman correlation coefficients for correlations between the interpersonal interaction questionnaire items and the Intrinsic Motivation Inventory scales. If a questionnaire item does not appear in the table, no correlations had a *P* value below .10 for that item.

Rater and Questionnaire Item		Interest/enjoyment		Effort/importance		Competence		Pressure/tension	
		CC^a^	*P* value	CC	*P* value	CC	*P* value	CC	*P* value
**Self-rating**									
	How much participant talks to other one	0.11	.39	-0.16	.22	0.07	.58	*-0.30*^b^	*.02*
	How much participant was talked to	0.11	.42	*-0.22*	*.097*	0.05	.70	*-0.38*	*.002*
	Game-relatedness of participant's statements	*0.31*	*.02*	*0.26*	*.05*	-0.09	.51	0.10	.44
	Overall valence	*0.25*	*.06*	-0.02	.90	0.09	.52	*-0.36*	*.004*
**Average of internal raters**									
	How much participant talks to other one	0.08	.53	-0.08	.55	0.18	.16	*-0.41*	*.001*
	How much participant was talked to	0.04	.79	-0.01	.91	0.13	.33	*-0.41*	*.001*
	Game-relatedness of participant's statements	0.17	.23	0.01	.94	*-0.29*	*.03*	*0.34*	*.01*
	Overall valence	*0.22*	*.097*	-0.04	.76	0.02	.90	*-0.36*	*.005*
**Average of external raters**									
	How much participant talks to other one	0.11	.44	-0.07	.62	*0.25*	*.08*	*-0.32*	*.02*
	How much participant was talked to	0.05	.71	-0.03	.81	0.05	.75	*-0.37*	*.008*
	Game-relatedness of participant's statements	0.13	.41	0.00	>.99	*-0.38*	*.01*	*0.33*	*.03*
	Overall valence	0.19	.19	0.08	.56	0.09	.52	-0.19	.19

^a^CC: correlation coefficient.

^b^Correlations with a *P* value below .10 are in italic.

**Table 3:** Spearman correlation coefficients for correlations between the interpersonal interaction questionnaire items and the Ten-Item Personality Inventory scales. If a questionnaire item does not appear in the table, no correlations had a *P* value below 0.1 for that item.

Rater and Questionnaire Item		Openness		Conscientiousness		Extraversion		Agreeableness		Neuroticism	
		CC^a^	*P* value	CC	*P* value	CC	*P* value	CC	*P* value	CC	*P* value
**Self-rating**											
	How much participant talks to other one	0.15	.25	0.03	.83	*0.29*^b^	*.02*	-0.08	.52	*-0.32*	*.01*
	How much participant was talked to	*0.21*	*.10*	0.02	.88	*0.21*	*.10*	-0.17	.19	*-0.31*	*.02*
	Valence of participant's statements	0.11	.42	*0.22*	*.097*	0.03	.83	*-0.22*	*.10*	*-0.29*	*.03*
	Game-relatedness of participant's statements	*-0.22*	*.10*	0.09	.50	*-0.24*	*.08*	-0.14	.29	0.10	.45
	Overall valence	0.20	.13	0.05	.70	0.18	.18	-0.12	.38	*-0.32*	*.01*
**Average of internal raters**											
	How much participant talks to other one	*0.29*	*.03*	0.05	.69	*0.30*	*.02*	-0.14	.27	*-0.34*	*.007*
	How much participant was talked to	*0.21*	*.10*	0.08	.53	*0.27*	*.03*	-0.10	.47	*-0.26*	*.04*
	Valence of participant's statements	0.19	.15	0.01	.97	-0.18	.18	-0.12	.38	0.05	.68
	Game-relatedness of participant's statements	*-0.30*	*.03*	-0.03	.85	*-0.28*	*.04*	-0.07	.63	0.21	.13
	Overall valence	0.14	.29	0.11	.42	0.17	.19	-0.19	.15	*-0.32*	*.01*
**Average of external raters**											
	How much participant talks to other one	*0.38*	*.006*	0.05	.74	*0.37*	*.009*	-0.20	.16	*-0.47*	*.001*
	How much participant was talked to	0.17	.25	0.05	.72	*0.31*	*.03*	-0.02	.92	-0.16	.27
	Valence of participant's statements	0.12	.43	-0.12	.43	-0.11	.46	-0.13	.40	0.03	.84
	Game-relatedness of participant's statements	*-0.30*	*.049*	0.06	.68	*-0.26*	*.09*	-0.07	.64	0.20	.19
	Overall valence	0.20	.17	0.12	.40	*0.31*	*.03*	0.00	.98	*-0.24*	*.10*

^a^CC: correlation coefficient.

^b^Correlations with a *P* value below .10 are in italic.

## Discussion

In this section, we first discuss the systematic differences between participants A and B observed on items 1-2 and 4-7, then discuss the results for different items of the questionnaire. We then discuss the correlations between the interpersonal interaction questionnaire, IMI, and personality scales. Finally, we discuss the need for further validation of our questionnaire with different populations and different game designs and briefly discuss possible alternative approaches to measuring conversation in serious games.

### Systematic Differences Between Participants

From an observer’s perspective, there should be little difference between the *A* participants and *B* participants, and items 1 and 2 should thus yield essentially the same ICC as should items 4 and 5 as well as 6 and 7. Although many ICCs were the same for these items, there were also notable differences—for example, ICCs for items 4 and 5 differ by as much as 0.27. We performed a follow-up analysis to determine whether the *A* participants were significantly different from the *B* participants but found no significant differences for age, gender, personality, or IMI. Thus, we believe that the difference in ICCs may be because of systematic differences in the study setup: different input devices (Bimeo for A and joystick for B) and different paddle positions on the screen (top for A and bottom for B). This possibility is supported by participants’ conversation (several commented on the differences between the Bimeo and joystick) and suggests that the reliability of the questionnaire may depend on the hardware used; however, the differences in ICCs may also be simply because of statistical noise, and this should be explored further.

### Amount of Conversation and Game Relatedness

On the basis of the ICCs for questions 1, 2, 6, and 7, we can conclude that (at least for this group of participants) observers can use our questionnaire to measure both the amount of conversation and its game relatedness very consistently, with both the internal and external raters exhibiting ICCs over .9. The most important clarifications for these items were what to do in cases of unbalanced conversation (1 person talks more than the other), what constitutes a 1 or 5, and whether metagame discussion should count as game related.

In addition, the raters sometimes asked how to compare coherent conversation (eg, focusing on a single topic for an extended amount of time) with incoherent conversation (eg, frequent grunts, “oops”es, “ohhh”s, and similar exclamations but few full sentences). In the end, we did not include explicit instructions regarding conversation coherence, although this could be addressed in a future update of the questionnaire—potentially even with additional items to measure this aspect. Another addition to the questionnaire could be a measurement of who takes the lead on the conversation: we observed several cases where both players talked about the same, but all periods of conversation were initiated by the same player even if the other player then contributed equally.

Finally, the participants were able to provide reasonably consistent ratings of their gameplay session, with ICCs between participants ranging from 0.51 to 0.82 for questions 1, 2, 6, and 7. The lower ICCs compared with observers can likely be attributed to the fact that participants are focused on playing the game throughout the session and thus do not keep track of their conversation to the degree that observers do.

### Conversation Balance

The item about conversation balance (item 3) exhibited relatively low ICCs compared with items 1 and 2. We believe that one major reason for this was the lack of actual imbalance in the dataset: most of the answers to this item were 1 or 2, whereas 4 occurred in only 1 pair, and 5 never occurred. Thus, the low ICCs for this item are likely because of a limited range of values. However, it is worth noting that the ICC between the participants was especially poor (0.27). As with items 1 and 2, this worse ICC is likely because of the difficulty of keeping track of conversation while playing the game.

Our opinion on this item is mixed. On the one hand, it is similar to items 1 and 2, and the same information should ideally be obtainable as the difference between those 2 items. However, in our dataset, there were several cases where raters gave the same answers to items 1 and 2, then indicated some conversational imbalance in item 3. In most of these cases, the raters’ reasoning was that there was not enough of an imbalance to warrant different answers for items 1 and 2 but enough of an imbalance to be noted in item 3. Thus, item 3 may be more sensitive to small imbalances than items 1 and 2. In the future, this item could be validated further by artificially introducing imbalanced conversations (thus determining if the lower ICCs in this study were because of a limited range of self-reported values). Alternatively, it may be possible to simply omit this item and modify items 1 and 2 so that they are more sensitive to small imbalances.

### Valence

The 2 items related to individual participants’ valence (4 and 5) exhibited relatively low ICCs: although most ICCs were between 0.4 and 0.6 (in the *fair* range), one was as low as 0.15. As with the balance item, we believe that these low ICCs were primarily because of a limited spread of values in our sample. Most pairs were rated as 3 or 4 on these 2 items, only 1 pair was rated as 2 on either item by any rater, and no pairs were rated 1. As a result of this limited spread, the ICCs are low despite good matches between raters: for example, the ICC between the 2 external raters is .49 for item 4, but those 2 raters gave the same answer to that item for 21 of the 25 independently rated pairs and never disagreed by more than 1 point on the 5-point scale.

The narrow spread of valence values is to be expected from a laboratory study, as participants do not wish to exhibit negative behavior when they know they are being observed and recorded on video. We believe that such negative behavior can be easily observed in real-world serious game environments, as several studies have documented very negative responses to competitive serious games [4,25]. Similarly, although few pairs were rated a *5* with regard to valence, we believe that this is also realistic for a laboratory study—a real-world answer of *5* would correspond to, for example, a therapist actively praising and verbally supporting a patient during exercise. The questionnaire could be validated for such extreme negative and positive valence ratings using, for example, actors, but we ultimately elected to simply acknowledge this limited evaluation, as we believe that the questionnaire is nonetheless valid and useful for cases of extremely positive or negative conversational valence.

On both items 4 and 5, the ICC between participants is higher than all other ICCs. We believe that this is because the participants have a better insight into their own valence than the raters do; although the raters have to determine valence based only on facial expressions and conversation, participants are largely aware of their own internal emotional processes. Furthermore, although raters sometimes had difficulty differentiating between honest statements, good-natured ribbing, and sarcasm or insults, the meaning of each sentence was likely clearer to the participants. In addition, ICCs involving video rater 2 were lower than the other ICCs. After a follow-up analysis, we believe that this is because video rater 2 was more likely than the other raters to rate pairs a *5* on items 4 and 5. We believe that this can be avoided in the future by more clearly emphasizing that extreme values should be used sparingly (as stated in the Rating instructions).

The item about overall valence (item 8) exhibited better ICCs than items 4 and 5—between 0.6 and 0.7. The reason for this difference is not entirely clear, although we believe that it is because this item had a somewhat greater spread compared with items 4 and 5. For example, there were several cases where raters gave a score of *4* on items 4 and 5 but a score of *5* on this item, with the justification “neither participant’s behavior was very positive on their own, but the overall mood was very positive.” We therefore believe that item 8 does provide useful data on its own and is not simply an average of items 4 and 5.

Finally, 1 way to potentially improve ICCs for valence items would be to provide more detailed instructions on how to analyze nonverbal behavior. Although our rating instructions included some examples on how to combine verbal and nonverbal behavior for purposes of rating valence, the raters commented that this was not always an easy task, and additional instructions may help produce more consistent ratings. In a longer version of the questionnaire, we could potentially even include separate items for verbal and nonverbal valence.

### Correlations With Intrinsic Motivation Inventory and Ten-Item Personality Inventory

Multiple correlations were observed between our questionnaire and the IMI (Table 2), confirming that conversation can provide insight into participants’ motivation in competitive serious games. Most notably, pressure/tension was negatively correlated with the amount of conversation as rated by both participants and internal and external raters, confirming preliminary findings obtained with unvalidated questionnaires [16]. Interestingly, however, correlations with the other 3 IMI scales differed among raters. First, a correlation between game relatedness and perceived competence was observed for internal and external raters but not the participants. Second, a correlation between overall valence and interest/enjoyment was observed for participants and internal raters but not external raters. Finally, correlations between game relatedness, interest/enjoyment, and effort/importance were observed only for participants.

For the Ten-Item Personality Inventory, all 3 groups’ (participants, internal raters, and external raters) ratings about the amount of conversation were correlated with openness, extraversion, and (negatively) neuroticism. Furthermore, all 3 groups’ ratings of overall valence were negatively correlated with neuroticism. This is not an unexpected result but does show that the amount and valence of conversation can differentiate between different groups of people, as preliminarily observed with unvalidated measures [23]. Interestingly, game relatedness ratings were negatively correlated with openness and extraversion, indicating that extraverted participants were more likely to chat with the other participants about other topics, whereas introverted participants were less likely to talk unless it is related to the game. However, similarly to the IMI, some correlations were only observed for some raters. For example, correlations between our questionnaire and agreeableness or conscientiousness were only observed for participants (but not observers); on the other hand, some correlations were only observed for observers but not the participants.

These differences in significant correlations between participants, internal raters and external raters are likely because of a mixture of statistical noise, perception biases, and additional insights. For example, as participants likely have a better insight into their own valence, their valence ratings are more likely to be correlated with personality and interest/enjoyment than the observers’ ratings. On the contrary, the fact that some correlations were not found for participants’ ratings (but were found for observers) may be because of differences in self-perception and perception of others. Although we cannot determine the reasons for these differences in detail, we believe that they should be considered when deciding whether to administer the questionnaire to participants. Furthermore, administering the questionnaire to participants and observers simultaneously may even allow explicit study of perception and bias in the context of competitive serious games.

### Further Validation: Target Populations for Serious Games

As the immediate next step, the questionnaire should be used in applied studies with actual target populations for serious games. This is a critical step for 2 reasons. First, participants who play serious games with an actual goal (eg, learning new skills) will likely exhibit a wider range of conversational valence than participants in a laboratory experiment, allowing better validation of the valence items. Second, the positive results observed in our study were obtained with a population of young unimpaired university students and are not guaranteed to generalize with other populations.

On the basis of our previous experience with serious games, we believe that our questionnaire would be directly usable with obese adolescents and adults (a common target population for exercise games [4,5]) as well as with older adults who use serious games to socialize [20] or maintain their cognitive abilities but do not have major cognitive impairments. Although such populations may, for example, talk less than the students evaluated in our study, the conversation would likely still be accurately measured by the questionnaire. Similarly, the questionnaire could still be used to measure the amount of conversation and game relatedness in people with, for example, chronic depression or reduced emotional expressivity, although valence ratings may be less reliable in such populations. However, the questionnaire may be significantly less reliable in populations with communication disorders (seen in, eg, cooperative games for language therapy [8]) or other cognitive impairments (seen in, eg, motor rehabilitation after stroke or traumatic brain injury, depending on the injury location). In such populations, all items of the questionnaire may be unreliable, and this should be evaluated in follow-up studies.

### Further Validation: Cooperative Games and Games With More Than 2 Players

Although results of our validation are promising, the questionnaire has only been tested with a 2-player competitive game. Cooperative serious games may involve different verbal interaction patterns that may reduce the reliability of the questionnaire. We believe that the questionnaire is general enough to apply to cooperative games, although mean values of different items may change—for example, because players may need to plan their actions for optimal cooperation, the game relatedness of the conversation may increase. However, we acknowledge that this needs to be verified with different cooperative game designs. A future version of the questionnaire could even include items that are specific to cooperative game designs, such as identifying leaders and followers based on the conversation.

The questionnaire also has not been tested with games for more than 2 players and includes items that refer to specific players. We believe that it could be easily expanded for 3- or 4-player serious games (suggested for, eg, language therapy [8]), but that significant modifications would need to be made for group games (seen in, eg, weight loss [4]). On the contrary, although the questionnaire was developed and validated for 2-player serious games played on a single computer, we believe that minimal modifications would be needed for Web-based gameplay (eg, telerehabilitation), entertainment games, or even for nongame tasks.

### Alternative Conversation Measures

Finally, although our questionnaire is designed to be brief and usable by both players and observers, we acknowledge that alternative measures may be able to obtain a more detailed or objective picture of the interpersonal interaction. One possible alternative would be to have video raters count the number of conversation instances as well as estimate each instance’s speaker, length, valence, and game relatedness. Although this would be time consuming and likely only feasible in offline analysis, it may provide additional details. Alternatively, automated audio analysis could be used to estimate the amount of conversation as, for example, the mean sound level recorded by each player’s microphone or the percentage of time that each player’s microphone sound level exceeds a certain threshold. Although this would likely not allow analysis of valence or game relatedness, it would provide a very objective measurement.

### Conclusions

Our brief measure of interpersonal interaction allows players and observers of a 2-player competitive serious game to rate the players’ amount of conversation as well as the conversation’s valence (positive or negative emotional content) and game-relatedness using a total of eight 5-point items. The amount of conversation and its game relatedness can be rated reliably, with ICCs over .9 for pairs of trained raters. Valence is more difficult to rate reliably, with ICCs between .5 and .7, but we believe that this is because of the limited range of valence values in our data (neutral to moderately positive) and that the brief measure could nonetheless be used to rate very negative or very positive conversations.

The questionnaire can be used to study user experience in competitive and cooperative serious games, which are becoming increasingly popular in fields such as rehabilitation and education. User experience with such games is known to depend on factors such as the player’s relationship with their coplayer, and a validated measure of interpersonal interaction will enable a better understanding of these factors, potentially leading to more efficient deployment of competitive and cooperative serious games. Furthermore, the questionnaire could be adapted for other applications such as entertainment games. However, we acknowledge that it has only been validated with healthy university students in a 2-player competitive serious game, and it should be further validated with different target populations for serious games (eg, stroke survivors) and with other game designs (eg, 2-player cooperation and group games).

## Appendix

Multimedia Appendix 1 Full interpersonal interaction questionnaire used in our study. This includes separate versions for observers and participants.

## Abbreviations

ICC: intraclass correlation coefficient
IMI: Intrinsic Motivation Inventory
