# Supplementary material for: A Brief Measure of Interpersonal Interaction for 2-Player Serious Games: Questionnaire Validation
Source: JMIR Serious Games. 2019 Jul 23;7(3):e12788. doi: 10.2196/12788 (PMC6683649; doi:10.2196/12788)
Supplement: Multimedia Appendix 1 [file games_v7i3e12788_app1.pdf]

Full questionnaire – Participant version

1. How much did you talk to the other player?

little to no talking				nearly constant talking
1	2	3	4	5

2. How much did the other player talk to you?

little to no talking				nearly constant talking
1	2	3	4	5

3. How balanced was the conversation? In other words, did both players talk about the same amount or did one player talk more than the other?

both players talked about the same amount		one player talked moderately more		one player dominated the conversation
1	2	3	4	5

4. How positive or negative were the things that you said during the game?

very negative				very positive
1	2	3	4	5

5. How positive or negative were the things that the other player said during the game?

very negative				very positive
1	2	3	4	5

6. Were the things that you said to the other player about the game or about other, unrelated topics?

mostly unrelated to the game				mostly related to the game
1	2	3	4	5

7. Were the things that the other player said to you about the game or about other, unrelated topics?

mostly unrelated to the game				mostly related to the game
1	2	3	4	5

8. How would you rate the overall conversation?

very negative				very positive
1	2	3	4	5

Full questionnaire - Observer version

1. How much did player A talk to player B?

little to no talking				nearly constant talking
1	2	3	4	5

2. How much did player B talk to player A?

little to no talking				nearly constant talking
1	2	3	4	5

3. How balanced was the conversation? In other words, did both players talk about the same amount or did one player talk more than the other?

both players talked about the same amount		one player talked moderately more		one player dominated the conversation
1	2	3	4	5

4. How positive or negative were the things that player A said during the game?

very negative				very positive
1	2	3	4	5

5. How positive or negative were the things that player B said during the game?

very negative				very positive
1	2	3	4	5

6. Were the things that player A said about the game or about other, unrelated topics?

mostly unrelated to the game				mostly related to the game
1	2	3	4	5

7. Were the things that player B said about the game or about other, unrelated topics?

mostly unrelated to the game				mostly related to the game
1	2	3	4	5

8. How would you rate the overall conversation between the players?

very negative				very positive
1	2	3	4	5
